# The Role of Trust as a Driver of Private-Provider Participation in Disease Surveillance: Cross-Sectional Survey From Nigeria

**DOI:** 10.2196/52191

**Published:** 2024-04-25

**Authors:** Ellen MH Mitchell, Olusola Adedeji Adejumo, Hussein Abdur-Razzaq, Chidubem Ogbudebe, Mustapha Gidado

**Affiliations:** 1 Mycobacterial Diseases and Neglected Tropical Diseases Unit Department of Public Health Institute for Tropical Medicine Antwerp Belgium; 2 Mainland Hospital Yaba Lagos Nigeria; 3 Department of Community Health and Primary Health Care Lagos State University Teaching Hospital Ikeja, Lagos Nigeria; 4 Health Research Unit Directorate of Planning, Research, and Statistics Lagos Ministry of Health Lagos Nigeria; 5 KNCV Tuberculosis Foundation Nigeria Abuja Nigeria; 6 KNCV Tuberculosis Foundation The Hague Netherlands

**Keywords:** surveillance, trust, Integrated Disease Surveillance and Response, IDSR, tuberculosis, notification, public-private mix, infectious disease, disease surveillance, surveillance behavior, health care worker, health professional, public health, Nigeria, survey, behavior, self-reported

## Abstract

**Background:**

Recognition of the importance of valid, real-time knowledge of infectious disease risk has renewed scrutiny into private providers’ intentions, motives, and obstacles to comply with an Integrated Disease Surveillance Response (IDSR) framework. Appreciation of how private providers’ attitudes shape their tuberculosis (TB) notification behaviors can yield lessons for the surveillance of emerging pathogens, antibiotic stewardship, and other crucial public health functions. Reciprocal trust among actors and institutions is an understudied part of the “software” of surveillance.

**Objective:**

We aimed to assess the self-reported knowledge, motivation, barriers, and TB case notification behavior of private health care providers to public health authorities in Lagos, Nigeria. We measured the concordance between self-reported notification, TB cases found in facility records, and actual notifications received.

**Methods:**

A representative, stratified sample of 278 private health care workers was surveyed on TB notification attitudes, behavior, and perceptions of public health authorities using validated scales. Record reviews were conducted to identify the TB treatment provided and facility case counts were abstracted from the records. Self-reports were triangulated against actual notification behavior for 2016. The complex health system framework was used to identify potential predictors of notification behavior.

**Results:**

Noncompliance with the legal obligations to notify infectious diseases was not attributable to a lack of knowledge. Private providers who were uncomfortable notifying TB cases via the IDSR system scored lower on the perceived benevolence subscale of trust. Health care workers who affirmed “always” notifying via IDSR monthly reported higher median trust in the state’s public disease control capacity. Although self-reported notification behavior was predicted by age, gender, and positive interaction with public health bodies, the self-report numbers did not tally with actual TB notifications.

**Conclusions:**

Providers perceived both risks and benefits to recording and reporting TB cases. To improve private providers’ public health behaviors, policy makers need to transcend instrumental and transactional approaches to surveillance to include building trust in public health, simplifying the task, and enhancing the link to improved health. Renewed attention to the “software” of health systems (eg, norms, values, and relationships) is vital to address pandemic threats. Surveys with private providers may overestimate their actual participation in public health surveillance.

## Introduction

Nigeria experienced five major infectious disease outbreaks during 2017, representing an unprecedented crisis for the public health system that laid bare many of the intersectoral collaboration gaps that hamper an effective public health response [1]. The existence of parallel reporting systems, authorities, and periods, along with variable case definitions challenge even the most well-intentioned and highly motivated health care workers to comply [2,3]. The globally networked, economically and culturally dynamic hub of Lagos, Nigeria, has long been identified as a place where timely information on emerging pathogens, pharmacovigilance, and infectious disease surveillance is crucial to the country’s public health [4].

Tuberculosis (TB) notification and cohort analysis are illustrative of the classical surveillance practice of a stigmatized condition worldwide and a bellwether of a country’s capacity for public health surveillance. Although TB service provision and reporting by the private sector is long recognized as an essential component of an effective TB program, it is still poorly theorized [5,6]. Many efforts to engage the private sector have been directive or transactional, driven by an incomplete or simplistic understanding of how private providers think [7]. The COVID-19 and mPox pandemics have heightened attention to the issues of trust and mistrust in public health authorities, whereas their centrality in TB surveillance has yet to be quantified [8,9].

Efforts to incentivize the private sector to render quality TB care and contribute to TB notification have intensified in recent years [10-13]. Many models are designed around logical inferences, but often without compelling evidence of efficacy to distinguish them [14]. Many public-private mix (PPM) models are transactional and/or directive, with a focus on resource transfer and regulatory oversight [7]. Although advocacy, additional professional society engagement, subsidized drugs, coordinating bodies, the introduction of advanced TB diagnostics, financial incentives, stricter penalties, and supervision interventions have been shown to improve notification initially, the gains are often modest and challenging to sustain [12]. Local stakeholders thus requested a study to generate insights into how to best set priorities among the diverse solutions and how best to distribute scarce drugs, diagnostics, staffing, and supervision resources.

**Figure 1:**
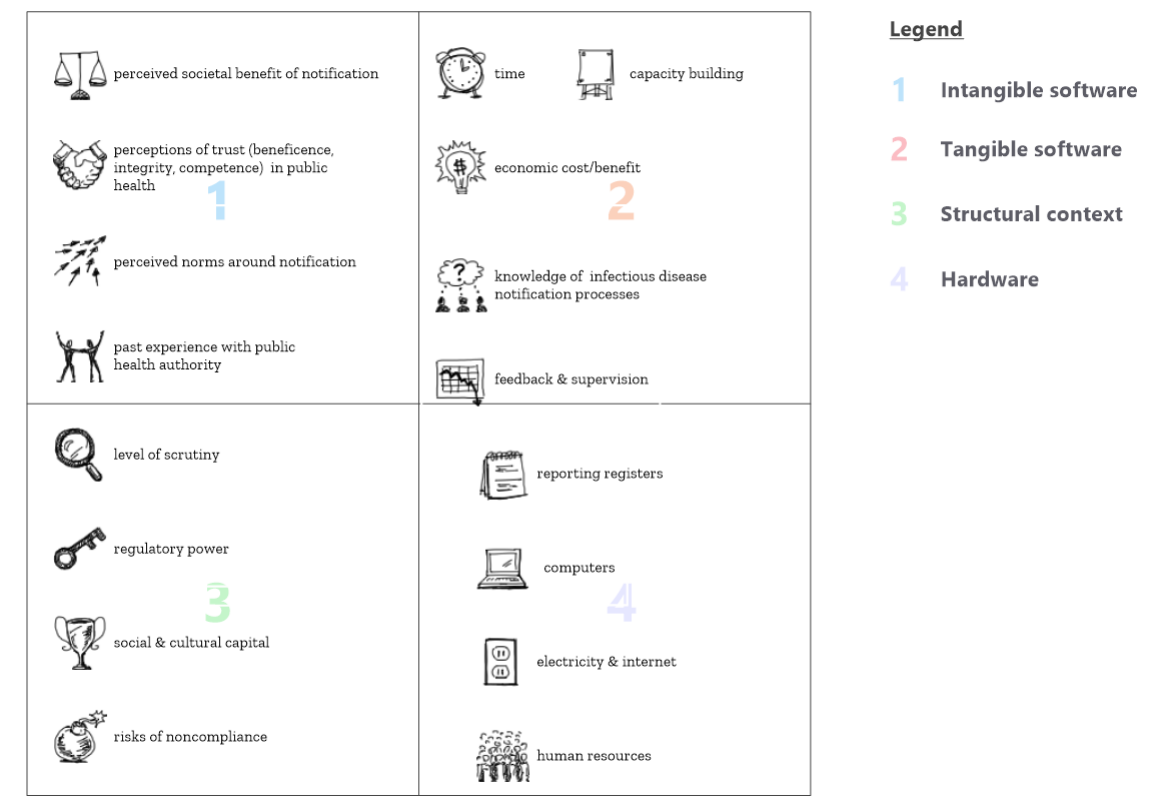
Hypothesized influences acting on infectious disease reporting by private providers.

We posited that the act of notifying a TB case is predicated on a set of expectations about systems, risks, rewards, penalties, and costs [15]. Surveillance assumes that specific types of patient information are available to be recorded (eg, test results and treatment outcomes) and that certain data formats (eg, registers and electronic platforms) exist that are accessible and intelligible. Notification obligations assume a certain knowledge of the legal and technical process of recording and reporting. Crucially, such an integrated approach assumes a set of values and willingness to contribute to surveillance as a public service [2]. For the desired public health participation to occur, potential contributors to systems may need to perceive the benefits as outweighing the risks [15-17]. As shown in Figure 1, we grouped the hypothesized influences on infectious disease reporting behavior into four categories: structural context, hardware, tangible software, and intangible software. This framework highlights how an enabling environment includes both mechanical and instrumental elements (ie, the “hardware”) and more relational or perspectival elements (ie, the “software”) [7,18].

Insights into the underlying reasons for facility noncompliance with surveillance requirements are needed if systems are to be reengineered in ways that make it worthwhile for providers to participate in TB notification. Indeed, early warning systems and antibiotic stewardship efforts are equally reliant upon the voluntary contribution of time-sensitive facility data. Mutual perceptions of competence, benevolence, and integrity form a crucial part of a surveillance system’s “software,” yet rarely receive as much focus as the “hardware” [7,18-20].

To improve private providers’ willingness to contribute to surveillance systems in Lagos, State Ministry of Health (SMOH) stakeholders sought assistance to answer the following policy questions: Given multiple systems for TB notification, how do private providers decide where and when to contribute their data to different surveillance systems? What are “unengaged” private providers’ concerns about notification? Is noncompliance with notification specific to TB or are they broadly noncompliant with infectious disease notification obligations? How does trust in the SMOH affect willingness to engage in public health surveillance?

The Nigerian Integrated Disease Surveillance and Response (IDSR) system is among one of the most dynamic and well-characterized surveillance systems in Africa [21]. Efforts to build and maintain rigorous surveillance systems to tackle emerging threats with pandemic potential have included policy, training, legislation, and validation exercises [3,22-28]. Over time, the system has evolved from a narrow Disease Surveillance and Notification Office (DSNO) to the comprehensive World Health Organization (WHO)–recommended IDSR of today.

The formal engagement of the Nigerian private sector in the PPM program for nationally recognized TB management commenced in 1993 [29]. PPM in Nigeria has been standardized, protocolized, and cautiously implemented by the national TB program [29,30]. Training and equipping health facilities with drugs and reagents were the most common forms of PPM engagement in Nigeria initially. Guidelines were updated approximately every 10 years. The use of incentives, performance-based finance, peer-led, and digital models have been employed and results have varied. Investments to increase private-provider collaboration in TB control in Lagos have been intense and involved multimethod approaches [12,30-34].

## Methods

### Study Design

We undertook a cross-sectional survey of the persons in charge of private health facilities without an ongoing relationship to the TB program, because these sites are considered to be those most likely to treat TB without reporting it. This assessment was part of a TB inventory study conducted to estimate the magnitude of underreporting [35,36]. Private facilities previously trained and equipped by the TB program were studied separately.

### Study Setting

Lagos State is in Southwest Nigeria, the commercial heart of Nigeria and home to the country’s most dynamic private sector. Although Lagos is the smallest state in Nigeria in terms of land mass, the population estimates range from 13 to 21.5 million. The population density exceeds 5000 individuals/km^2^, roughly 25 times the national average population density of 226 individuals/km^2^. More than 65% of Lagos’ population lives below the poverty line. Lagos has 2.5 private health facilities per 10,000 people, making it one of the more complex health systems of any megacity [37]. The health facilities open, operate, and then cease operations or move locations dynamically [37]. Faith-based and private not-for-profit health facilities represent a smaller proportion (1.7%) of the Lagosian health sector than in other Nigerian states [38].

### Sampling

Probability proportional to size sampling was conducted among private health facilities with no documented engagement with the TB program. Private facilities provided with TB registers and trained by the national TB program were recruited for a parallel study with distinct aims. Full details on health facility sampling, pilot testing, data collection, data management, and quality assurance have been described previously [35].

### Recruitment

Potential participants were contacted by telephone for recruitment appointments. Written information and letters from the TB program and ethical review board were shared to establish the factual basis of the visit.

One interview was conducted at each of the participating facilities. Inclusion criteria for survey respondents were two-fold: (1) health care workers with influence over the completion of TB case notification and (2) able to provide individual informed consent (eg, over 18 years of age). Semistructured interviews were administered on site using paper questionnaires by trained interviewers (see Multimedia Appendix 1).

At “unengaged” private facilities, the following 10 issues were explored: (1) awareness of the mandatory reporting of TB in Nigeria; (2) perceived trustworthiness, competence, and beneficence of the SMOH; (3) attitude toward specific aspects of TB notification processes; (4) attitude toward specific PPM engagement incentives (clinical training, drugs, reagents); (5) self-reported participation in the State TB & Leprosy Control Program (STBLCP) and/or the IDSR, also known as the DSNO; (6) self-reported challenges with disease reporting via IDSR (closed-ended); (7) self-reported reasons for nonreporting diseases (closed-ended); (8) self-reported participation in either reporting system (STBLCP or IDSR); (9) self-reported reasons for nonreporting diseases (closed-ended); (10) willingness to engage (and prior TB engagement experiences) with the STBLCP.

### Study Measures

The scale for Citizen Trust in Government Organizations (CTGO), a validated 11-item scale of public perceptions toward government institutions, was adapted to capture Nigerian private practitioners’ trust in public health and disease surveillance systems [39]. The CTGO scale measures three dimensions: (1) perceived competence of public health authorities, indicating the extent to which a provider perceives a (government) organization to be capable, effective, skillful, and professional (4 items); (2) perceived benevolence, indicating the extent to which a private provider perceives a (government) organization to care about the welfare of the public and to be motivated to act in the public interest (3 items); and (3) perceived integrity, indicating the extent to which a provider perceives a (government) organization to be sincere, tell the truth, and fulfill its promises (4 items). Pilot testing of the questionnaire occurred in 5 facilities. Cronbach α and the intraclass correlation coefficient of the scale were used to gauge validity and internal consistency. Survey items with poor construct validity during pretesting were deleted. Survey items were reduced from a total of 76 to 53 and worded via piloting to improve validity and acceptability.

SPSS (IBM) version 25 and the R *psych* package were used for statistical analyses. The magnitude and variance of the responses were examined to identify central tendencies and outliers were considered for further exploration. Data were summarized as percentages, means, and medians, and Student *t* tests were used to compare mean scores. For statistical tests, *P*<.05 was considered statistically significant. A regression model was developed to predict the binary outcome of infectious disease case notification using IDSR.

### Ethical Considerations

The study protocol was reviewed and approved by the Health Research and Ethics Committee of the Lagos State University Teaching Hospital (registration number 04/04/2008). Participation was voluntary and providers could consent to zero, partial, or full patient data access. Noncompliance with notification obligations was kept confidential and is described in ways to preclude deductive disclosure. After the data sets were linked, all personal and geographic identifiers were removed. A small monetary incentive (US $5) was offered for participation.

## Results

### Sample Characteristics

There were 278 representatives surveyed from private facilities that did not report TB. They ranged in age from 21 to 81 years, with a mean age of 46 (SE 0.8) years and an average of 18.2 (SE 0.8) years of clinical practice. Among the 278 respondents, 62.9% (n=175) were men and 36.3% (n=101) were women. Among the private facilities represented, 40.6% were at the primary level, 53.6% at the secondary level, and 4.7% unclassified; 94.6% (n=263) of the facilities were for-profit and 5% (n=14) were faith-based (see Table 1). Among the total 294 representatives contacted, 278 (84.2%) consented to participate. The recruited sample of health care workers was in line with the intended sample in terms of the total sample size, local government area distribution, and facility level [35].

**Table 1:** Sociodemographic characteristics of participants (N=278).

Variable			Value
**Age group (years), n (%)**			
	<30	26 (9.4)	
	30-39	58 (20.9)	
	40-49	53 (19.1)	
	50-59	73 (26.3)	
	>60	67 (24.1)	
	No response	1 (0.4)	
**Age (years)**			
	Mean (SD)	46.3 (12.9)	
	Range	12-81	
**Gender, n (%)**			
	Man	175 (62.9)	
	Woman	101 (36.3)	
	No response	2 (0.7)	
**Type of practice, n (%)**			
	General practitioner	247 (88.7)	
	Specialist	31 (11.2)	
**Facility level, n (%)**			
	Primary	113 (40.6)	
	Secondary	149 (53.6)	
	Unspecified	13 (4.7)	
	No response	3 (1.1)	
**Type of facility, n (%)**			
	For-profit	263 (94.6)	
	Faith-based	14 (5.0)	
	No response	1 (0.4)	
**Years of practice, n (%)**			
	<10	79 (28.4)	
	10-19	47 (16.9)	
	20-29	58 (20.9)	
	>30	58 (20.9)	
	No response	36 (12.9)	

### Knowledge of Obligations and Self-Reported Infectious Disease Reporting Behavior

As shown in Table 2, over three-quarters of the respondents were aware of the obligation to notify TB cases. A minority (13.5%) reported having been notified a disease recently (within weeks of the survey), 58.2% had last reported a disease within months of the survey, and 28.4% last reported it within years of the survey. Among those surveyed, nearly one-quarter reported on-site capacity to diagnose TB (Table 2). 

**Table 2:** Participants’ behaviors, challenges, and recommendations for improved disease notification in Lagos, Nigeria.

Variable			Respondents, n (%)
Facility had capacity to diagnose TB^a^ (n=276)			203 (73.6)
Facility had capacity to provide TB treatment (n=276)			126 (45.7)
Aware of obligation to report TB (n=277)			215 (77.6)
Evidence of provision of TB treatment in the last 12 months (N=278)			33 (11.9)
**Most recent notification (n=141)**			
	Weeks ago	19 (13.5)	
	Months ago	82 (58.2)	
	Years ago	40 (28.4)	
Comfortable reporting TB patients to the LGA^b^ (n=267)			242 (90.6)
Facility has ever notified about TB (n=258)			132 (51.2)
**Entity notified (n=151)**			
	IDSR^c^ (DSNO^d^)	74 (49.0)	
	TB program	75 (49.7)	
	Can’t remember	2 (1.3)	
State TB program ever offered TB training (n=270)			63 (23.3)
Any training by another TB organization (n=272)			37 (13.6)
**Self-reported monthly IDSR (DSNO) notification (n=261)**			
	Always	128 (49.0)	
	Sometimes	35 (13.4)	
	Never	98 (37.5)	
**Frequency of challenges to IDSR (DSNO) notification (n=187)**			
	Seldom	116 (62.0)	
	Often	33 (17.6)	
	Don’t know	38 (20.3)	
**Type of challenges in notification**			
	Lack of time to fill out forms (n=146)	35 (24.0)	
	Unavailability of forms (n=136)	20 (14.7)	
	No data to fill out forms (n=136)	15 (11.0)	
	Form design very confusing (n=135)	10 (7.4)	
**Suggestions to improve the IDSR (DSNO) notification (n=62)**			
	Provide more training in notification	15 (24.2)	
	Make reporting electronic	11 (17.7)	
	Supportive supervision	11 (17.7)	
	Simplify forms	9 (14.5)	
	Provide feedback	2 (3.2)	
	Provide incentives	2 (3.2)	

^a^TB: tuberculosis.

^b^LGA: local government area.

^c^IDSR: Integrated Disease Surveillance Response.

^d^DSNO: Disease Surveillance and Notification Office.

The vast majority (90.6%) of respondents reported being hypothetically comfortable with notifying TB patients to the local government. In contrast to high levels of comfort with notification as a norm, only about half (51.2%) reported ever having notified a TB case. Of those who self-reported ever having notified a TB case, roughly half (49.0%) reported doing so via the IDSR (DSNO) system, while 49.7% reported doing so via the TB program and 1.3% could not recall which system they used. Among the 33 health facilities with records of treating 156 TB cases, none had been notified [36]. Self-reported participation in disease notification and disease surveillance varied and did not often align with the findings of independent verification of notification by the facility [36].

**Figure 2:**
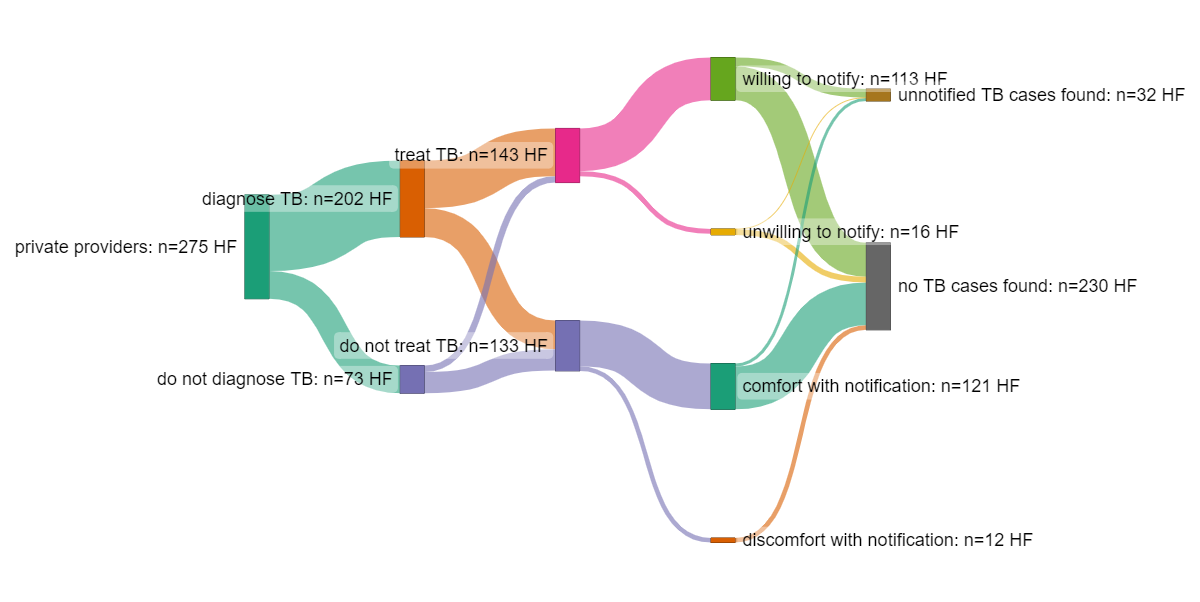
Comparison of notification attitudes and self-reported notification behavior versus presence of notifiable TB cases. HF: health facility; TB: tuberculosis.

As shown in Figure 2, a majority of unnotified TB cases (31/33) were discovered in the records of health facilities of respondents who self-reported comfort with notification. TB cases were also found in the records of health facilities where respondents stated that they lacked the capacity to treat TB (8/23).

Self-reported notification behavior differed according to sociodemographic characteristics. Older health care workers with more experience were more likely to self-report comfort with notification of TB cases, having previously notified of a case, and participation in monthly disease surveillance. Women reported less comfort with notification than men (87.2% vs 92.4%) and were significantly less likely to report “always” contributing to monthly surveillance (41.5% vs 52.7%) (Table 3).

**Table 3:** Health care worker self-reported participation in disease surveillance by sociodemographic characteristics (N=276).

Characteristic		Total	As a private medical practitioner, I am comfortable in notifying TB^a^ cases to the local government		This facility has previously notified a TB case to any government entity		This facility provides monthly reports to DSNO^b^ using the 003 form		
							Always	Sometimes	Never
**Gender, n (%)**									
	Men	175 (63.9)	158 (92.4)		81 (50)	87 (52.7)		13 (7.9)	65 (39.4)
	Women	101 (38.1)	82 (87.2)		50 (53.2)	39 (41.5)		22 (23.4)	33 (35.1)
Age (years), mean (SE)		46.3 (0.8)	47.0 (0.9)		49.2 (1.1)	50.4 (1.1)		41.1 (2.1)	43.0 (1.4)
Years of health care practice, mean (SE)		18.2 (0.8)	18.9 (0.9)		21.9 (1.2)	22.2 (1.1)		13.2 (2.5)	14.8 (1.3)

^a^TB: tuberculosis.

^b^DSNO: Disease Surveillance and Notification Office.

### Perceived Competence, Benevolence, and Integrity of Public Health Authorities

The adapted trust items had a Cronbach α of 0.91, suggesting good internal consistency. However, the scale showed ceiling effects and a bimodal distribution, necessitating reciprocal transformation (see Figure S1 in Multimedia Appendix 2). Initially, the gender difference in trust was not explained by age or years of experience (see Figure S2 in Multimedia Appendix 2). After transformation, the distribution of the trust scores varied by gender and self-reported notification behavior; men tended to report more trust and more monthly IDSR notification behavior than women (Figure S3 in Multimedia Appendix 2).

**Figure 3:**
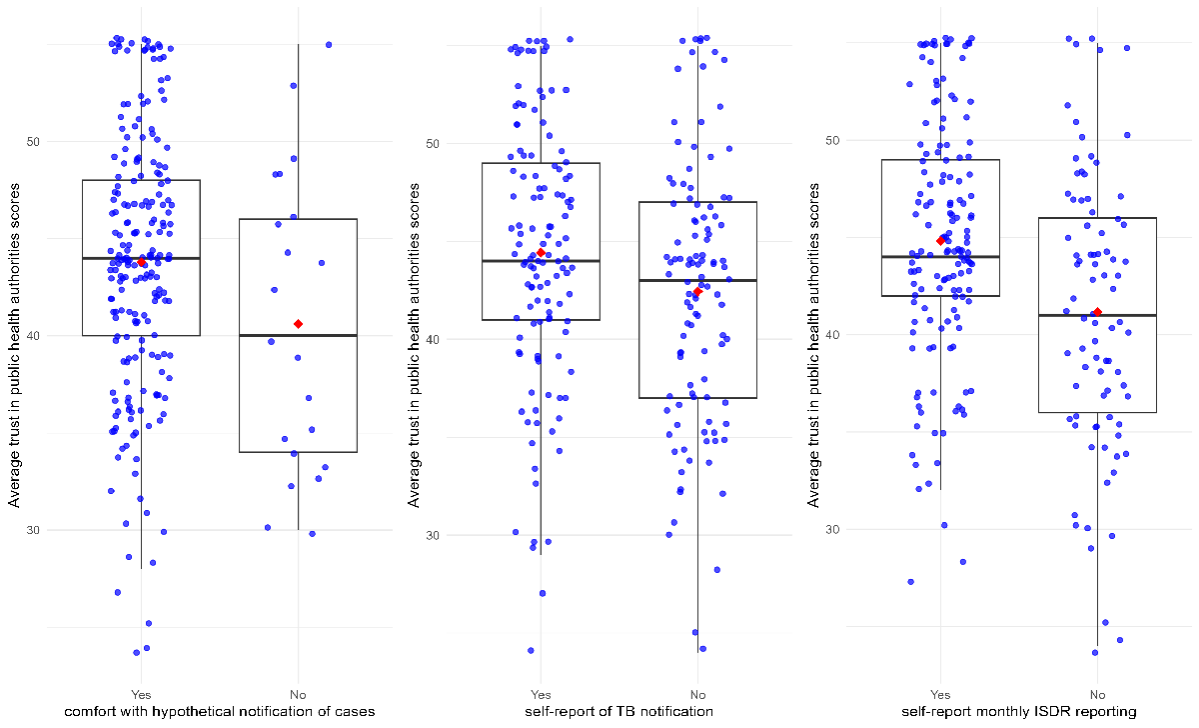
Comparison of mean trust scores by self-reported notification intention and behaviors (N=278). IDSR: Integrated Disease Surveillance Response; TB: tuberculosis.

Neither the trust scale nor the subscales (competence, benevolence, and integrity) were normally distributed according to the Kolmogorov-Smirnov test. The Nigerian public health authority trust scale had a 3-factor structure with loading of four competence items, three benevolence items, and four integrity items (see Figure S4 in Multimedia Appendix 2). Scores on the trust scale ranged from 11 to 55, with higher values implying greater trust. The median value was 44 (IQR 39-48) (Table 4). A minority of health care workers expressed doubts about the trustworthiness of the SMOH to conduct disease surveillance (Figure 3). Private providers who were not comfortable notifying TB scored the SMOH lower on the benevolence subscale of trust. Health care workers who affirmed always notifying via IDSR monthly reported higher median trust in the state’s public disease control capacity and had higher median scores on all three subscales compared to those of health care workers who indicated never reporting (Table 4).

The minority of private providers who reported being uncomfortable reporting TB cases to the state were also less likely to report that the SMOH was benevolent, acting in their interests (Table 4). Private providers who reported that they did not participate in the IDSR monthly reporting system were slightly less likely to report that the SMOH was competent and effective in providing health services (91% vs 98%; *P*=.03) and were less likely to view the SMOH as a capable regulatory agency (81% vs 95%; *P*=.02). Nonparticipants in disease surveillance reported lower median scores of SMOH competence, benevolence, and integrity than those who reported “always” submitting monthly reports. There were no significant differences in attitudes among those who reported ever notifying a TB case and those who did not (Figure 3).

Among those who were not comfortable notifying TB as mandated (n=156), the reasons for discomfort with disease notification included practical, logical, strategic, and economic concerns. The most common reason (58/156, 28.9%) given for noncompliance was lack of access to the surveillance “hardware” (eg, notification forms and registers). Approximately one-quarter of TB providers were doubtful that the low volume of TB patients they treated in their facility merited mastery of the TB notification forms and procedures. In addition, approximately 16% of the respondents incorrectly believed that notification to the TB program was unnecessary if they participated in the IDSR system (Table 5).

**Table 4:** Private providers’ attitudes toward State Ministry of Health trustworthiness according to tuberculosis (TB) and Integrated Disease Surveillance Response (IDSR) reporting behavior.

Scale item	As a private medical practitioner, are you comfortable in notifying your TB patients to the local government?				Self-reported monthly IDSR notification behavior^a^		
	Yes (n=242), median (95% CI)	No (n=25), median (95% CI)	*P* value	Always (n=198), median (95% CI)		Never (n=98), median (95% CI)	*P* value
Private providers’ trust in public health authorities (reciprocal transformation)	0.023 (0.023-0.023)	0.025 (0.023-0.029)	.02	0.022 (0.022-0.023)		0.024 (0.023-0.025)	.001
Perceived competence subscale	4.5 (4.0-5.0)	4.0 (4.0-7.0)	.11	5.0 (5.0-6.0)		4.0 (4.0-5.0)	<.001
Perceived benevolence subscale	3.0 (3.0-4.0)	2.0 (1.0-3.0)	<.001	3.0 (3.0-4.0)		3.0 (3.0-4.0)	.02
Perceived integrity subscale	4.0 (4.0-6.0)	2.0 (0.0-4.0)	.12	4.0 (4.0-6.0)		2.0 (2.0-4.0)	.02

^a^Participants responding “sometimes” were classified as missing.

**Table 5:** Private providers’ rationales for discomfort with tuberculosis (TB) notification (n=156).

Reason^a^	Respondents, n (%)
I lack access to TB forms and/or TB registers	58 (28.9)
The number of TB patients treated here is too small to merit mastery of so many TB forms and procedures	31 (19.5)
Notifying TB to the state TB program is unnecessary because TB cases are reported to IDSR^b^	26 (15.9)
Notification does not align with my business interests	19 (12.0)
My reputation for patient confidentiality would be at risk	16 (10.2)
Lack of time to fill out reporting forms	14 (8.9)
I am unconvinced of the purpose/value of TB notification	12 (7.6)
TB forms and TB registers are confusing and complex	11 (7.1)
Reporting TB cases is not required in this state	8 (5.2)
Supervision by the TB program would be a burden	7 (4.5)

^a^Respondents could give more than one answer.

^b^IDSR: Integrated Disease Surveillance Response.

## Discussion

### Principal Findings

Private providers who did not notify TB cases via the disease surveillance system were younger and more likely to doubt the competence and effectiveness of public health authorities. Younger health care workers and women perceived fewer incentives to notify and indicated many obstacles to compliance. The summary of our hypothesis testing results is provided in Table S1 of Multimedia Appendix 2.

In contrast to the conclusion of a 2018 rapid assessment of TB surveillance in Nigeria, our findings show that over three-quarters (77.6%) of the “unengaged” providers fully understood that TB notification is required by law. More than half (51%) had notified TB in the past [40]. However, some private providers seemed confused by the seemingly duplicative notification mandates of the IDSR and the STBLCP.

A plurality of Lagos providers remained skeptical of the value of notification to the SMOH. Providers had low levels of trust in the state’s disease control and surveillance efforts. Our study demonstrates that lower trust scores correlate with lower participation. Such a perceived lack of effectiveness and regulatory competence of Nigerian state public health authorities is not without precedent. Oleribe et al [16,17] also found that clinicians throughout Nigeria reported limited faith in the governmental commitment to public health and health care workers. Uchenna et al [3] identified a “bad attitude” as a barrier to completion of IDSR in Enugu state. Lafond et al [26] showed that providers often lack confidence that notification would make a positive difference. Our study extends this body of work by showing that the “software” of public health in Nigeria (ie, the values, norms, relationships, power, and mutually defined aims) are associated with mission-critical behaviors of key actors.

The development of trust needs reciprocal strengthening. Nigerian TB program stakeholders also express ambivalence regarding the competence, integrity, and motives of the private for-profit sector in TB care [29,41]. Distrust in the ethics and altruism of private providers is similarly well documented [42]. In India, Nair et al [43] found that lack of trust on the part of health authorities was a barrier to public-private TB collaboration. Some policy makers assume that private practitioners only respond to financial incentives; however, the evidence for this hypothesis is often overstated [41,44-46]. While providers who participated in this study reported a willingness to notify in exchange for free or subsidized drugs, diagnostic commodities, and patient materials, they also expressed support for nonmonetary incentives. Peer norming and social network recognition can be powerful forces in the health professions [44,45]. A majority (57.9%) of Lagos private providers stated that they would contribute to TB notification if they received professional recognition of their contributions from their medical peers and they were certain their peers were also engaged. Implementation research on these lower-cost peer-norming means to improve behavior is needed.

This study sought to understand attitudes toward participation in infectious disease surveillance using TB notification as an illustration. A unique feature of the study is the holistic measurement of complex governance trust constructs such as public health competence, benevolence, and integrity using validated scales.

Private provider “noncompliance” with public health obligations is a complex, multicausal behavioral phenomenon. Providers are influenced by peer norms around recording and reporting but also harbored doubts about the purpose and value of participation in public health surveillance. In contrast to earlier studies, Nigerian private providers did not lack knowledge of the notification obligation, nor did transactional “engagement” prompt full compliance. Although 17% of the respondents stated that electronic systems would be favored, 1 in 10 private providers had concerns about safeguarding patient confidentiality, which may impact their willingness to partner in WhatsApp groups, notification apps, and electronic registers.

In India, Thomas et al [47] found that patients’ confidentiality concerns (24%) and fear of offending patients (11%) were barriers to notification. El Emam et al [15] also found that private providers worry that disclosure of case counts could have adverse legal, ethical, financial, and regulatory consequences.

Effective engagement of private sector providers in Lagos and elsewhere will require mutual trust building, compromise, and respect. Achieving this will involve trade-offs, especially early on. Greater attention to end-user acceptability and the design of surveillance systems is paramount. While women were more likely to state that they were uncomfortable with disease reporting, none of the 156 TB cases found in the 33 facilities had been notified, highlighting the limits of PPM surveys as a method to understand notification behavior. Going forward, ethnographic research and inventory studies are needed to explore the gap between the rhetoric of self-reported notification behavior and actual notification behaviors.

As private providers’ motivations and TB capacity-building needs varied widely, offering a menu of incentives and enablers to this heterogeneous group would be a strategic approach to gain broad compliance. Some of the strategies that are being trialed to engage private sites include continuing medical education credits, vouchers for subsidized rapid molecular testing, and computerized chest-x-ray imaging, among others. Although these traditional PPM incentives (eg, training; free informational, educational, and communication materials; medical commodities; and free diagnostic tests) make sense for private providers with substantial TB caseloads, for small practitioners who will only ever treat limited TB patient volumes, sustainable options might include simpler, less-onerous, anonymous notification systems [2].

Simultaneous PPM initiatives funded by the Global Fund, United States Agency for International Development, Directorate-General for International Cooperation of the Netherlands, and the Centers for Disease Control and Prevention were implemented in Lagos during 2017-2020 [2,12,34,41,48]. Simplified private-sector TB surveillance systems have been introduced, including an Android app (STARRTB) and peer-to-peer WhatsApp notifications [13]. Efforts to improve the interoperability and integration of the IDSR and STBLCP TB notification systems have also been implemented by the WHO [2,13,49]. Although no interventions have yet addressed the issues of perceived regulatory effectiveness of public health agencies explicitly, efforts to streamline and simplify reporting burdens for private providers could contribute to increases in the perceived beneficence of public health agencies.

Nationally, the proportion of TB notifications by private providers increased from 11% in 2015 to 22% in 2021, but the addition of multiple reporting modalities raises the possibility of double counting and complicates attribution [2,12,13,41,48]. In the National Strategic Plan of the TB program, an additional US $35 million was foreseen for improving reporting in 2022. Continued appraisal of the return on investment of these diverse strategies is warranted.

One of the critical hurdles in gaining private-sector cooperation in antibiotic stewardship, surveillance, pandemic response, and other vital public health efforts is building the reputation of state public health institutions as credible, competent, and committed stewards of data. Training and deploying of apps are necessary, but likely insufficient to substantially and sustainably increase notification in the long term [13]. A possible strategy to build trust in the SMOH should be part of any approach to boost stakeholders’ motivation to comply with notification obligations. Timely provision of valid scientific information and appreciation of the complementary strengths of private providers can help ministries of health earn the respect of private providers. Few contributors to the IDSR in Nigeria receive regular feedback or are aware of how the data are used [50]. Going forward, this “software” of surveillance needs as much attention as the equipment and human resources required to perform it.

Sensitivity on the part of TB policy makers will be required to craft a minimalist TB surveillance system that is easy to use and appropriately concise to be acceptable to reluctant providers. Private providers are numerous, but their individual TB caseloads tend to be small; therefore, it is unsurprising that they may not wish to invest time in mastery of the complex TB registers common in infectious disease surveillance. This is particularly true when providers are unsure how their TB data are to be used or whether public health gains accrue to their communities via participation in these systems. A plurality of private providers (49%) stated that they are willing to contribute to a TB notification system if the data submitted were anonymous. As unique IDs are instituted via electronic recording and reporting, the necessity of the collection of patient names should be revisited. Ways to satisfy providers’ anonymity preferences for case notification should be explored [15].

An infectious disease surveillance system acceptable to private providers would collect fewer variables, report less frequently, and integrate the task with the existing IDSR obligations. TB stakeholders at the national and international levels would need to be willing to accept less granular information from the private sector in exchange for higher adherence, fidelity, and completeness. Surveillance systems based on semitrusted partners that protect privileged and proprietary information are possible [15].

### Limitations

Given the sensitivity of the topics in the study, the methods have certain caveats and design choices that need to be taken into consideration when interpreting the findings. Scales to detect social desirability were not included. A bimodal distribution with ceiling effects was observed; use of a structured survey did not allow us to probe all underlying rationales for noncompliance and the model did not explain all variances. Survey refusals were more common among facilities that treated TB but did not notify it (22% vs 4%), suggesting that the findings may underrepresent the full diversity of rationales for noncompliance. Sampling quotients overestimated TB treatment provision in the unengaged private sector, meaning that the majority (88%) of those interviewed were not faced with decisions about whether or not to notify TB cases and thus their responses may reflect historical or hypothetical choices. However, a strength of the study design is that it measured both providers’ *self-reported reporting behavior* and their *actual* reporting behavior so that socially desirable response bias is revealed in the juxtaposition as a finding.

This exploratory study endeavored to adapt and test a scale of public health authority trust as a possible contributor to understanding infectious disease surveillance behavior. While the trust scale proved robust, trust alone was insufficient to explain the variability in notification behavior. Mistrust in public authorities proved difficult to disentangle from mistrust of surveys. Additional methodological innovation may be required to overcome the influences of social desirability and acquiescence. The greater mistrust reported by those with more seniority may be confounded by the greater candor afforded by stature.

### Conclusion

New forms of public-private collaboration in surveillance are needed that align with the varied interests of private providers, reflecting their varied caseloads and capacity for recording and reporting. While spurring desired provider behavior may seem a matter of assembling an enticing package of carrots and sticks, achieving this is demonstrably difficult without addressing the underlying governance and trust considerations that blunt private providers’ willingness. Moreover, our study suggests that it may be more effective to adapt the TB notification system to make it more responsive to end-user needs than to modify providers’ attitudes and behavior. The lessons learned are relevant for the design of other surveillance systems, including postmarketing pharmacovigilance of new health technologies, patient safety reporting, antibiotic stewardship, and early warning systems for emerging pathogens.

## Appendix

Multimedia Appendix 1 Survey Instrument.

Multimedia Appendix 2 Distribution of CTGO scores according to monthly IDSR (Figure S1); scatter plot of trust according to years of experience in the health care field by gender (Figure S2); distribution of public authority trust scores according to gender and self-notification behavior (Figure S3); factor structure of the adapted trust in public health authorities scale (Figure S4); hypothesis test summary (Table S1). CTGO: Citizen Trust in Government Organizations; IDSR: Integrated Disease Surveillance Response.

## Abbreviations

CTGO: Citizen Trust in Government Organizations
DSNO: Disease Surveillance and Notification Office
IDSR: Integrated Disease Surveillance and Response
PPM: public-private mix
STBLCP: State Tuberculosis and Leprosy Control Program
TB: tuberculosis
WHO: World Health Organization

## Data Availability

Deidentified data are available for research purposes upon request to the corresponding author.
