# Supplementary material for: The Role of Trust as a Driver of Private-Provider Participation in Disease Surveillance: Cross-Sectional Survey From Nigeria
Source: JMIR Public Health Surveill. 2024 Apr 25;10:e52191. doi: 10.2196/52191 (PMC11082728; doi:10.2196/52191)
Supplement: Multimedia Appendix 1 [file publichealth_v10i1e52191_app1.docx]

***Questionnaire for “UNENGAGED” PRIVATE FACILITIES***

L*ocation: private health facility*

*Staff to be interviewed: treating clinician AND person responsible for notification*

***Dear clinician, We request you to kindly provide the following details***

1. Type of Practice
2. Location of practice:
   1. Latitude: _______
   2. Longitude: ___________
3. Do you have the capacity to diagnose tuberculosis among your clients in this facility?

- Yes
- No
- I do not know/recall

1. Do you provide TB treatment to patients in this facility when your patients request it?

- Yes
- No
- I do not know/recall

Now we would like to ask you some questions about the state Ministry of Health and their role in disease surveillance.

|  | **Strongly agree** | **Agree** | **Neither agree nor disagree** | **Disagree** | **Strongly disagree** |
| --- | --- | --- | --- | --- | --- |
| 1. Overall, the State Ministry of Health is a capable and proficient in conducting disease surveillance. |  |  |  |  |  |
| 1. The State Ministry of Health is competent and effective in providing health services. |  |  |  |  |  |
| 1. In general, The State Ministry of Health is very knowledgeable about disease control. |  |  |  |  |  |
| 1. The State Ministry of Health performs its role of regulating health services very well. |  |  |  |  |  |
| 1. If I required help, The State Ministry of Health would do its best to help me to notify TB cases. |  |  |  |  |  |
| 1. I believe that the State Ministry of Health would act in my best interest. |  |  |  |  |  |
| 1. The State Ministry of Health is interested in my effectiveness as a health care provider, not just its own. |  |  |  |  |  |
| 1. The State Ministry of Health is truthful in its dealings. |  |  |  |  |  |
| 1. The State Ministry of Health is sincere and genuine. |  |  |  |  |  |
| 1. If a contract is made, the State Ministry of Health would keep its commitments. |  |  |  |  |  |
| 1. I would characterize The State Ministry of Health as honest. |  |  |  |  |  |

1. Before our contact with you, were you aware of the obligation to report TB patients treated to the state?

o Yes

o No

o I don’t know

1. *If Yes to question 16*, when was the last time you notified any government entity of a mandatory disease

………………………. Specify in weeks/Month/Year.(Circle the appropriate one).

1. As a private medical practitioner, are you comfortable in notifying your TB patients to the local government?

o Yes

o No

**Rationales for non-compliance with obligatory infectious disease reporting**

What are some of the reasons that you are uncomfortable with disease notification *(tick all that apply)*

| 1. Notification does not align with my business interests | **Sp** |
| --- | --- |
| 1. Reporting TB cases is not required in this state |  |
| 1. Lack of time to fill out reporting forms |  |
| 1. Notifying TB to the State TB program is unnecessary because TB cases are reported to DSNO (IDSR) |  |
| 1. I am unconvinced of the purpose/value of TB notification |  |
| 1. Supervision by the TB program would be a burden |  |
| 1. I lack access to TB forms and/or TB registers |  |
| 1. TB forms and TB registers are confusing, complex |  |
| 1. The number of TB patients treated here is too small to merit mastery of so many TB forms and procedures |  |
| 1. My reputation for patient confidentiality would be at risk |  |
| 1. Others please specify |  |

1. Has this facility ever a notified TB case to any government entity before?

o Yes

o No

*If No skip to question x*

1. To which government entity was the notification made?
   1. DSNO (IDSR)
   2. TB program

**TB engagement- Recruitment experiences**

1. Has the State TB program ever offered to provide TB training, TB medicines, or reagents for TB diagnostic tests to this facility?

- Yes
- No
- I do not know/recall

1. Has any other TB organization (e.g. ARFH, KNCV, FHI) provided TB training, TB registers, TB drugs, reagents for TB diagnostic tests?

- Yes
- No
- I do not know/recall

1. If an offer of engagement was made and rejected, what were the reasons for not participating in the public-private partnership for TB? ___________________

**Disease notification**

1. Does this facility provide monthly reports to DSNO (IDSR) using the 003 form?

- *Yes, always*
- *Yes, sometimes*
- *No* (If No to question 35 skip Questions 36 – 38)
- I don’t know – unfamiliar with Form 003
- I don’t know – unfamiliar with reporting practices of facility

1. How does the information arrive at the DSNO (IDSR) at the LGA?

- Electronically
- By courier
- By telephone
- DSNO (IDSR) collects
- Other: (specify)
- I don’t know

1. How often do you experience challenges in completing the DSNO (IDSR) notification process?

- Seldom
- Often
- I don’t know

1. What are the challenges you experienced in completing the DSNO (IDSR) process? (tick all that apply)

- Lack of time to fill out all the data required
- DSNO (IDSR) forms were not available (provided)
- DSNO (IDSR) forms design is confusing, complex
- Data to fill DSNO (IDSR) forms is not always available.
- Others please specify

**Preferred solutions**

1. What might be done to overcome or improve the DSNO (IDSR) notification system to make it easier for facilities like you to report cases of disease?

I would be motivated to record and report information about my TB patients to the state TB program if……

|  | **Strongly disagree** | **disagree** | **Don’t know** | **agree** | **Strongly agree** |
| --- | --- | --- | --- | --- | --- |
| 1. If I received training in the use of TB forms and registers |  |  |  |  |  |
| 1. If I was certain my peers were doing it also. |  |  |  |  |  |
| 1. If I did not need to reveal the names of my patients |  |  |  |  |  |
| 1. If I did not need to be visited by the TBLS |  |  |  |  |  |
| 1. If I did not need to fill out all the variables, but rather and abbreviated form |  |  |  |  |  |
| 1. If I could send the data by phone (SMS) |  |  |  |  |  |
| 1. If I were surveyed by phone twice a year |  |  |  |  |  |
| 1. If I provided the data to HEFAMMA instead of the State |  |  |  |  |  |
| 1. If I received clinical training on TB in exchange for TB notification |  |  |  |  |  |
| 1. If I received free TB drugs for my patients in exchange |  |  |  |  |  |
| 1. If received free diagnostic test reagents for my patients in exchange |  |  |  |  |  |
| 1. If I received professional recognition of my contributions from my medical peers |  |  |  |  |  |
| 1. If I received medical commodities, such as test kits |  |  |  |  |  |
| 1. If I receive patient education materials |  |  |  |  |  |
