# Supplementary material for: The Role of Trust as a Driver of Private-Provider Participation in Disease Surveillance: Cross-Sectional Survey From Nigeria
Source: JMIR Public Health Surveill. 2024 Apr 25;10:e52191. doi: 10.2196/52191 (PMC11082728; doi:10.2196/52191)
Supplement: Multimedia Appendix 2 [file publichealth_v10i1e52191_app2.docx]

**Multimedia Appendix 2**

The trust scores had ceiling effects (see Figure S1) so reciprocal transformation was required.

**Figure S1: Distribution of CTGO scores according to Monthly IDSR**


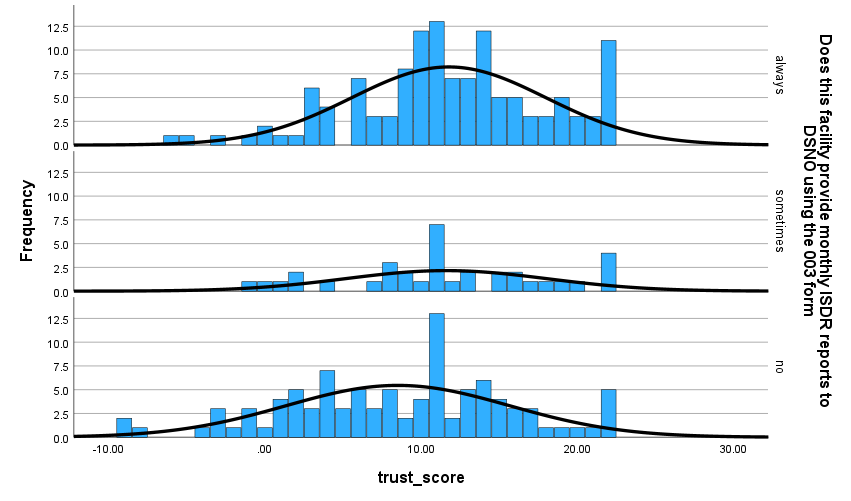


Neither age nor experience explained the gender difference in trust scores as shown in Figure S2.

**Figure S2: A scatter plot of trust according to years of experience in the healthcare field by gender**


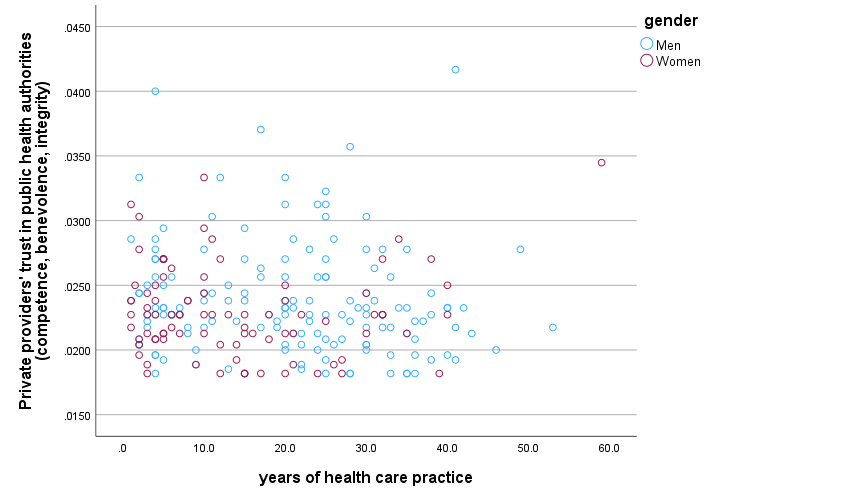


To mitigate the observed ceiling effects due to possible social desirability and/or acquiescence bias, trust scores were reciprocally transformed. After transformation the distribution of the trust scores varies by gender and self-reported notification behavior. Men tended to report more trust and more monthly IDSR notification behavior than women (Figure S3). This analysis does not account for hierarchical differences (e.g. roles) within the private facility.

**Figure S3:**


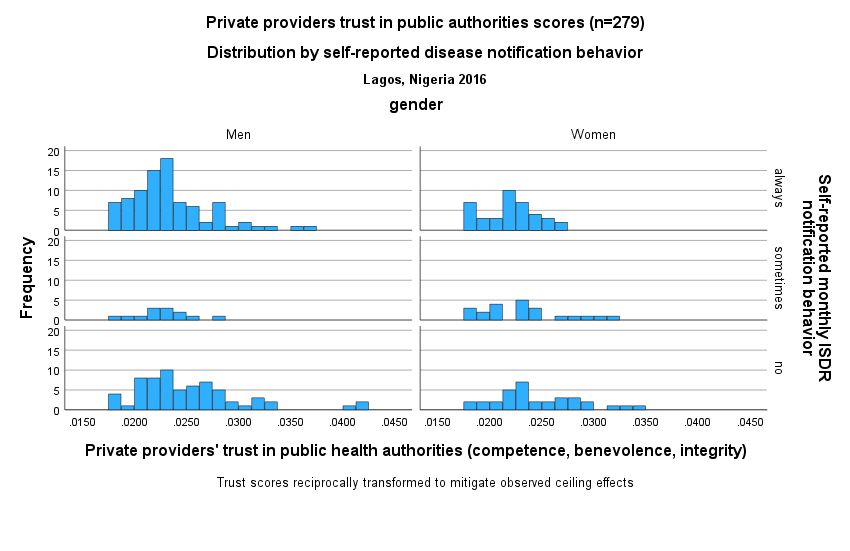


The factor structure of the adapted trust in public health authorities scale reflects the three-factor structure of the original CTGO (See Figure S4).


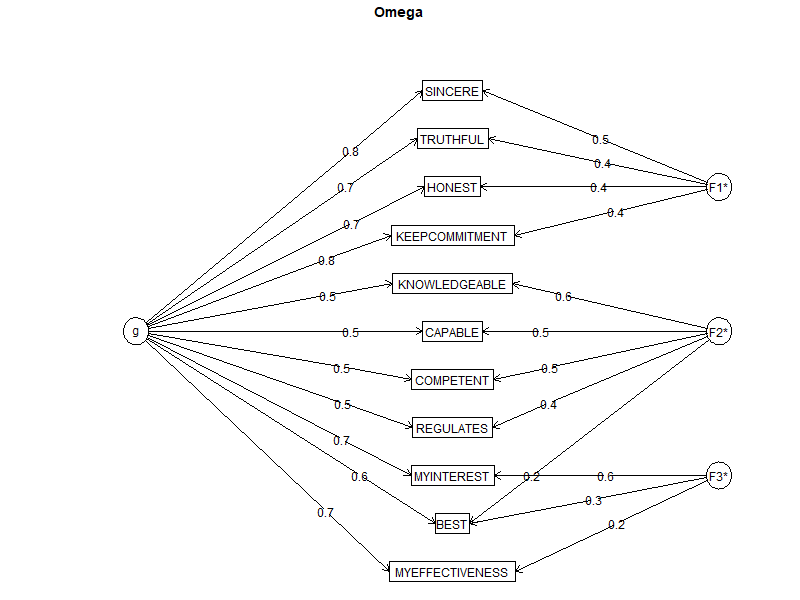


Neither the trust scale nor the sub-scales (competence, benevolence, integrity) were normally distributed according to the Kolmogorov-Smirnov Test.

| **Table S1: Hypothesis Test Summary** | | | | |
| --- | --- | --- | --- | --- |
|  | Null Hypothesis | Test | Sig.^a,b^ | Decision |
| 1 | The distribution of competence_rescale is normal with mean 4.59 and standard deviation 2.61789. | One-Sample Kolmogorov-Smirnov Test | <.001 | Reject the null hypothesis. |
| 2 | The distribution of benevolence_rescale is normal with mean 2.99 and standard deviation 2.16075. | One-Sample Kolmogorov-Smirnov Test | <.001 | Reject the null hypothesis. |
| 3 | The distribution of integrity_rescale is normal with mean 2.97 and standard deviation 3.10243. | One-Sample Kolmogorov-Smirnov Test | <.001 | Reject the null hypothesis. |
| 4 | The distribution of Trust in public health authority (competence, benevolence, integrity) is normal with mean 10.55 and standard deviation 6.83753. | One-Sample Kolmogorov-Smirnov Test | <.001 | Reject the null hypothesis. |
| 5 | The distribution of Trust_scale_imput is normal with mean 43.55 and standard deviation 6.83753. | One-Sample Kolmogorov-Smirnov Test | <.001 | Reject the null hypothesis. |
| 6 | The distribution of Private providers' trust in public health authorities (competence, benevolence, integrity) is normal with mean .0236 and standard deviation .0041865. | One-Sample Kolmogorov-Smirnov Test | <.001 | Reject the null hypothesis. |
| a. The significance level is .050. | | | | |
| b. Lilliefors Corrected. Asymptotic significance is displayed. | | | | |
